# Physiological stress markers during breath‐hold diving and SCUBA diving

**DOI:** 10.14814/phy2.14033

**Published:** 2019-03-25

**Authors:** Marion Marlinge, Mathieu Coulange, Richard C. Fitzpatrick, Romain Delacroix, Alexie Gabarre, Nicolas Lainé, Jennifer Cautela, Pierre Louge, Alain Boussuges, Jean‐Claude Rostain, Régis Guieu, Fabrice C. Joulia

**Affiliations:** ^1^ C2VN INSERM INRA Aix‐Marseille Université (AMU) Marseille France; ^2^ APHM: Assistance Publique des Hopitaux de Marseille Marseille France; ^3^ Department of Hyperbaric Medicine Sainte Marguerite University Hospital Marseille France; ^4^ School of Medical Sciences University of New South Wales Sydney New South Wales Australia; ^5^ Laboratory of Biochemistry Timone University Hospital Marseille France; ^6^ Department of Cardiology North Hospital Marseille France; ^7^ HIA Saint Anne Toulon France; ^8^ UFR STAPS Université de Toulon La Garde France

**Keywords:** Apnea, biological stress markers, copeptin, cortisol, hypoxemia, SCUBA

## Abstract

This study investigated the sources of physiological stress in diving by comparing SCUBA dives (stressors: hydrostatic pressure, cold, and hyperoxia), apneic dives (hydrostatic pressure, cold, physical activity, hypoxia), and dry static apnea (hypoxia only). We hypothesized that despite the hypoxia induces by a long static apnea, it would be less stressful than SCUBA dive or apneic dives since the latter combined high pressure, physical activity, and cold exposure. Blood samples were collected from 12SCUBA and 12 apnea divers before and after dives. On a different occasion, samples were collected from the apneic group before and after a maximal static dry apnea. We measured changes in levels of the stress hormones cortisol and copeptin in each situation. To identify localized effects of the stress, we measured levels of the cardiac injury markers troponin (cTnI) and brain natriuretic peptide (BNP), the muscular stress markers myoglobin and lactate), and the hypoxemia marker ischemia‐modified albumin (IMA). Copeptin, cortisol, and IMA levels increased for the apneic dive and the static dry apnea, whereas they decreased for the SCUBA dive. Troponin, BNP, and myoglobin levels increased for the apneic dive, but were unchanged for the SCUBA dive and the static dry apnea. We conclude that hypoxia induced by apnea is the dominant trigger for the release of stress hormones and cardiac injury markers, whereas cold or and hyperbaric exposures play a minor role. These results indicate that subjects should be screened carefully for pre‐existing cardiac diseases before undertaking significant apneic maneuvers.

## Introduction

SCUBA diving and apneic diving (breath‐hold diving) both involve immersion, increased hydrostatic pressure, physical activity, and cold exposure. They are different in that apneic dives are hypoxic (and hypercapnic), whereas SCUBA dives are hyperoxic through hyperbaric inspired air and often high O_2_ concentration N_2_/O_2_ mixes. Thus, there are similarities and differences in the mechanisms by which these different dives could physiologically stress the body.

SCUBA diving, while breathing air, is a physiological stressor through cold exposure, increased hydrostatic pressure, and hyperbaric inspired O_2_. SCUBA dives have been reported to induce increased levels of stress hormones prolactin (Anegg et al. 2002) and cortisol related to immersion depth (Zarezadeh and Azarbayjani 2014). These dives have been associated with transient endothelial dysfunction (Culic et al. 2014) likely mediated through hyperbaric O_2_, and have been linked to reduced cognitive performance (Pourhashemi et al. 2016) and stress cardiomyopathy (Baber et al. 2016). On the other hand, improved well‐being and reduced stress have been reported recently in recreational SCUBA divers (Beneton et al. 2017).

Spearfishing competitions and recreational apneic diving commonly involve 4–5 h of immersion with repeated voluntary dynamic apneic phases that can represent more than 2 h of apnea and, in temperate climates, the water is often cold. Apneic hypoxia combined with endurance physical activity, cold exposure, and increased hydrostatic pressures can exert intense physiological stresses on divers. For example, a release of S100B, a marker of cerebral stress, has been described after a single apnea followed by a black out (Linér and Andersson 2009) or preceded by hyperventilation and glossopharyngeal insufflations (Andersson et al. 2009) suggesting a brain stress during breath hold diving. However, such a release of S100B has not been observed after a maximal static apnea (Kjeld et al. 2015) suggesting that this cerebral stress could also be related to other factors than hypoxia. Exposition to high pressure of dives was known to induce brain injury (Tamaki et al. 2010), hemoptysis, and pneumomediastinum (Henckes et al. 2011) and is often associated with increased plasma markers of oxidative stress (Theunissen et al. 2013).

Despite the popularity of spearfishing and breath‐hold diving worldwide, the stress effects of repeated voluntary apnea are poorly understood and require investigation. Commonly, voluntary apnea has been studied through the cardiovascular adaptation of the diving reflex (Bergman et al. 1972; Andersson et al. 2004), which protects the heart and brain against hypoxia (Breskovic et al. 2011). However, the triggers, release and actions of stress hormones during breath‐hold diving are poorly understood. Accurately measured levels of stress hormones and cardiac markers could provide a better understanding of unexplained mishaps during breath‐hold diving.

Cortisol is the marker most commonly used to assess the stress response. Copeptin, the C‐terminal domain of antidiuretic hormone (ADH or vasopressin), is an early stress marker of neuro‐hypophysial origin and short half‐life. It is nonspecific but marks severe stress (Zhang et al. 2014), particularly during cardiovascular dysfunction (Reichlin et al. 2009). Markers of striatal muscle injury (lactate, myoglobin), cardiac injury (brain natriuretic peptide (BNP), troponin), and hypoxia (ischemia‐modified albumin: IMA) (Bar‐Or et al. 2001) also provide measures of physiological stress.

This study examines sources of physiological stress in diving as revealed by circulating stress markers. We compared responses to three conditions; (1) dry static apnea (hypoxia), (2) apneic dives (hypoxia, pressure, cold, activity), and (3) SCUBA dives (hyperbaric O_2_, pressure, cold, activity). To identify additional stress effects of hydrostatic pressure, cold and activity from hypoxic stress effects, we compared apneic dives with dry static apnea performed on land at rest. To identify the relative stresses of hypoxic and hyperoxic conditions, we compared apneic dives with SCUBA dives. We hypothesized that the combined stress factors of cold and pressure exposure associated with physical activities during SCUBA and apneic dives would be more stressful compared to the hypoxia strictly induced by a long static apnea.

## Materials and Methods

### Subjects

Thirty‐six volunteers recruited from three populations participated to this experiment. Twelve men (mean age 37 ± 8 years) who were experienced apneic divers were recruited during the French Spearfishing Championship (Saint Raphael, July 2014). Twelve men (mean age 51 ± 10 years) who were experienced SCUBA divers were recruited from the military diving school of St Mandrier, France. Eight men (mean age 48 ± 4 years) who were not divers were recruited from departmental staff to serve as a control group. Participant characteristics are summarized in Table 1. All subjects were nonsmokers, without medication, inflammatory, cardiovascular, or respiratory disease. Subjects gave written informed consent prior to the study, which was approved by the Aix‐Marseille University Human Research Ethics (South Marseille) and conducted in line with the principles of the Declaration of Helsinki.

**Table 1:** Characteristics of the subjects and study parameters

	CNT	APN	SCU
Subjects characteristics			
Age (years)	48 ± 4 [34–63]*	37 ± 8 [25–53]	51 ± 9 [32–60]*
Body mass (kg)	79 ± 14 [63–89]	77 ± 10 [60–100]	75 ± 11 [65–90]
Height (cm)	176 ± 11 [169–189]	178 ± 9 [172–190]	175 ± 6 [170–183]
Experience (years)		16 ± 11 [2–40]	21 ± 14 [11–24]
Study parameters			
Diving			
Dives (count)		65 ± 10 [70–110]	1
Depth (m)		15 ± 10 [5–40]	40 ± 10 [21–63]
APN‐D total apnea (min)		80 ± 20 [77–197]	
Static			
APN‐S apnea duration (s)		340 ± 105 [140–450]	

Values are Mean ± SD [Range] measured in Controls (CNT), apneic group (APN), and SCUBA dive group (SCU).

APN‐D total apnea corresponds to the sum of apnea dives done by each participant of APN group during the 5 h spearfishing competition and recorded by a dive computer. APN‐S apnea duration corresponds to the duration of the single dry submaximal apnea done by each participant of APN group a week before the spearfishing competition.

* Indicates significant difference at *P* < 0.05 *between APN‐SCU groups and APN‐CNT groups* (Mann–Whitney U test).

### Protocol

Four studies were undertaken. The practicalities of the different situations in each study required some adaptation of the timing protocol used to measure stress markers.

#### Study 1: Apnea static (APN‐S)

The 12 experienced apnea divers performed a dry static apnea (APN‐S) protocol between 07:00 and 09:00. This protocol has been previously used on elite apnea divers (Joulia et al. 2013, 2015). Briefly, subjects rested supine and breathed normally in ambient room conditions (25± 2°C) for 10 min. Blood samples were then collected for baseline levels of stress markers. To increase the diving reflex response (Hentsch and Ulmer 1984), subjects then performed five short static voluntary apneas of 90–180 sec separated by 1‐ to 5‐min rest periods (individual usual warm‐up protocols). To prevent hyperventilation, subjects were instructed to breathe as quietly as possible before commencing a breath‐hold. Five minutes after this warm‐up protocol, subjects performed a submaximal static breath‐hold that ranged from 140 to 450 sec (Table 1). Two blood samples were collected immediately and 10 min after the apnea breaking point. Participants were not ask to perform their maximal performance in order to minimize the risk of a black out that would have jeopardized their participation to the championship taking place the next week.

#### Study 2: Apnea dive (APN‐D)

These 12 apneic dive subjects were also tested the day of the French National Championship, which was 1 week after these subjects had participated in the static apnea testing (Study 1). A baseline blood sample was drawn at rest in the supine position within 2 h of commencing competition. A second sample was drawn while supine 10–15 min after the end of competition. The competition lasted 5 h during which each subject wore an insulating diving suit (5 mm thick) and dive computer (SUUNTO D4, Italy) to monitor the dive parameters (Table 1). Surface water temperature was 23°C, immersion depths were between 5 and 40 m, and the total apnea durations (i.e., the sum of each apnea dive done during the 5 h competition) were between 77 and 197 min. These values show that the level of physical activity during the competition was quite intense. Furthermore, the average dive speed (100 m/min) calculated from the dive profiles recorded on the participant's computer (i.e., twice the depth divided by the sum of descending and ascending durations) also argue in favor of an intensive physical exercise.

#### Study 3: SCUBA dive (SCU‐D)

For these 12 participants, blood samples for stress markers were collected on the day of a SCUBA dive. Baseline samples were collected while supine after 10 min of rest while breathing normally in ambient room conditions (22 ± 2°C). They then performed a single professional dive with air breathing wearing the insulating diving suit (5 mm) and computer to monitor dive parameters (Table 1). Dive time was recorded as the time at maximal depth. Mean dive duration was 21 min (range 11–24 min), mean depth was 15 m (range 5–40 m), and mean water temperature was 17°C (range 15–19°C). Blood samples were collected 10–20 min after the end of the dive.

#### Study 4: Control levels (CNT)

A control group, including eight sedentary men, was used to verify that the SCUBA and apneic groups did not present at rest a significant difference in stress marker concentrations in relation to their specific practice. Blood samples were collected at rest in the supine position after 10 min of rest while breathing normally in room air. Mean air room temperature was 24°C (range 20–25°C).

### Blood sampling

Capillary blood samples (500 *μ*L) were collected by digital puncture. Blood analysis was performed using the EPOC^®^ system (ALERE, Jouy en Josas, France) on 90 *μ*L for hematocrit, hemoglobin, and lactate measurement. A 400 *μ*L sample was centrifuged immediately and the supernatant stored in ice until delivery to the laboratory for measurement of copeptin, albumin, cortisol, and cardiac markers. Arterial blood oxygen saturation (SpO_2_) was measured with a pulse oximeter (NPB 40: Nelcor Puritan Bennett, California) affixed to the left median finger.

### Measurement and analysis

#### Copeptin

As described previously (Bourgeois et al. 2013), copeptin was measured using the BRAHMS Copeptin US KRYPTOR assay (Thermo Fisher Scientific, Waltham, Massachusetts) that uses a monoclonal antibody, europium cryptate, and laser excitation at 337 nm (detection threshold: 1 pmol/L; inter or intraassay variation: <10%; normal range: <13 pmol/L).

#### Albumin and C‐reactive protein

The biomarkers albumin and C‐reactive protein (CRP) were measured using the Cobas 8000^®^ apparatus (Roche, Zurich, Switzerland) for ultrasensitive CRP (usCRP) (detection threshold: 0.05 mg/L; intraassay variation: <10%; normal range: <5 mg/L).

#### Cardiac troponin I, myoglobin, and brain natriuretic peptide (BNP) measurement

Cardiac troponin I (cTnI) and myoglobin were measured using the ADVIA Centaur^®^ apparatus (Siemens, Erlangen, Germany). Ultrasensitive cTnI detection threshold was 0.006 *μ*g/L; the intraassay variation <10% in 0.006–50 *μ*mol/L range; normal values <0.05 *μ*g/L. Normal values for myoglobin are <110 *μ*g/L. BNP was measured using a two‐site sandwich immunoassay (range: 2–50 ng/L; intraassay variation: <10%; normal range: <200 ng/L).

#### Ischemia‐modified albumin (IMA)

IMA measurement was performed as previously described using the albumin Cobalt binding test (Nee et al. 2013). Briefly, samples were incubated with Co^++^, which binds to the albumin N‐terminus. After washing, the proportion of albumin that binds Co^++^ is inversely correlated with the quantity of IMA (Bar‐Or et al. 2001; Joulia et al. 2015). Results are expressed in arbitrary units (AU: normal range: <100 AU).

### Statistics

Quantitative variables, expressed as means ± standard deviations, were compared using nonparametric statistical tests. Differences between two groups were tested using the Mann–Whitney *U* test, whereas within‐group differences were tested using the Wilcoxon signed rank test for paired samples (2‐tail). Basal differences between the three groups were tested using a Kruskal–Wallis ANOVA. When a group effect was observed, a Mann–Whitney U test was performed to compare groups 2 by 2. Differences were considered as significant when *P* < 0.05 after Bonferroni correction for multiple comparison. Statistical analyses were performed using Statistica 6 (StatSoft Inc., Oklahoma).

## Results

Every subject completed all test protocols. No cardiovascular or neurological events occurred in any apneic or SCUBA test. No significant differences were observed between biomarker basal values for the three groups (Kruskal–Wallis Chi^2^_(*n* = 32, dL = 18)_ = 22.63, *P* = 0.21) indicating that, despite their training, the measured stress markers were not more elevated at rest in the apnea divers or SCUBA divers compared to control group.

### Ischemia‐modified albumin and copeptin

**Figure 1:**
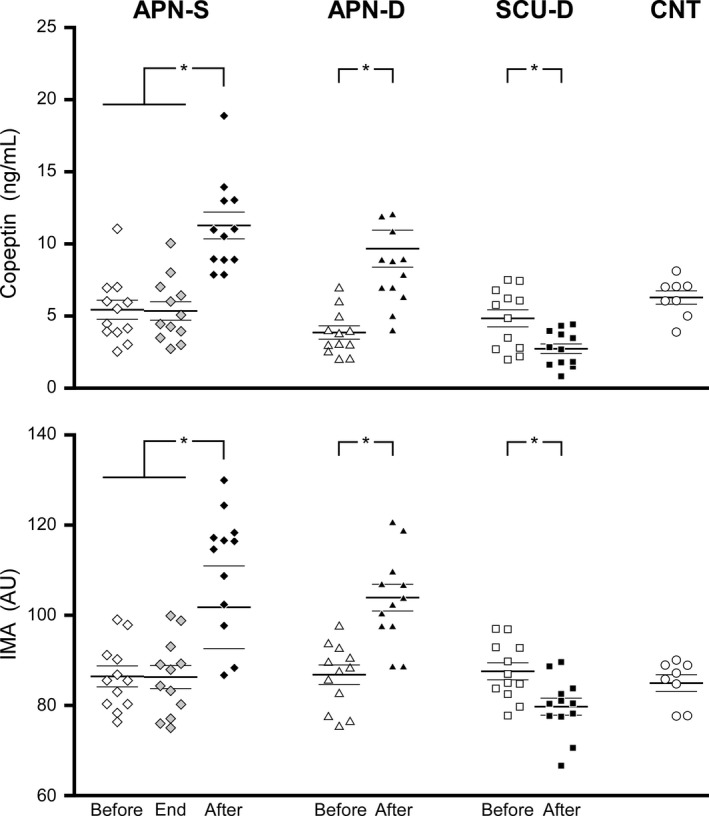
Individuals and group (average – thick horizontal bar ± 1SD – thin horizontal bars) values of copeptin (upper panel) and IMA (lower panel) measured before (open symbols) and 10 min after (black symbol) dry static apneas (APN‐S), apneic dives (APN‐D), and SCUBA dives (SCU‐D). The APN‐S situation was the only one in which values could also be measured exactly at the end of the apnea (gray symbols). All basal values (before) were compared with the values at rest of a control group (CNT). *Indicates a significant difference at *P* < 0.05 between the values measured before and after the experimental situation.

After the apneic dive (APN‐D), IMA levels were 19.4% greater than baseline (*n* = 12; *W* = 2.93; *P* = 0.0033 Fig. 1A) and copeptin levels 155% greater than baseline (*n* = 12, *W* = 3.06, *P* = 0.0022, Fig. 1). These were not significantly different from level changes induced by dry static apnea (APN‐S) of a 26% increase in IMA (*n* = 12, *W* = 0.667, *P* = 0.504) and 108% increase in copeptin (*n* = 12, *W* = 0.152, *P* = 0.88) at the equivalent time of 10 min post apnea (Fig. 1). Note in Figure 1 (APN‐S), that IMA and copeptin levels sampled immediately after this apnea were not different to baseline and the increase was not evident until 10 min later. For the scuba dive (SCU‐D), the opposite response was observed. IMA and copeptin levels both decreased by 8.7% (*n* = 12, *W* = 2.93, *P* = 0.0033, Fig. 1) and 34% (*n* = 12, *W* = 2.59, *P* = 0.0096, Fig. 1), respectively, after the single dive.

### Others blood parameters

The apneic dive (APN‐D) induced significant increases in plasma cortisol (*n* = 12, *W* = 2.82, *P* = 0.005), cardiac troponin I (*n* = 12, *T* = 0, *Z* = 3.062, *P* = 0.0022), brain natriuretic peptide (*n* = 12, *W* = 3.06, *P* = 0.0022), hemoglobin (*n* = 12, *W* = 2.932, *P* = 0.0033), hematocrit (*n* = 12, *W* = 2.85, *P* = 0.0044), myoglobin (*n* = 12, *W* = 2.82, *P* = 0.005), and albumin (*n* = 12, *W* = 2.59, *P* = 0.01). There was no significant change in any of these measures after the SCUBA dive (SCU‐D) or after the static apnea (Table 2). A decrease in pulse oximetry Sp0_2_ was only observed at the end of the dry static apnea APN‐S protocols (mean ‐15%, *n* = 12, *W* = 3.062, *P* = 0.0022). Note that the delay in obtaining SpO_2_ levels in the APN‐D and SCU‐D conditions allowed recovery to normal levels.

**Table 2:** Biomarkers, before and after experimental protocols

Markers	CNT	APN‐S		APN‐D		SCU‐D	
		Before	After	Before	After	Before	After
Cortisol (*μ*mol/L)	0.21 ± 0.16	0.2 ± 0.05	0.21 ± 0.05	*0.2* ± *0.07*	*0.35* ± *0.09* *	0.18 ± 0.12	0.21 ± 0.12
Copeptin (ng/mL)	6.62 ± 1.9	*5.4* ± 2.3	11.2 ± 3.2*	3.8 ± 2.2	9.7 ± 4.5*	6.02 ± 2.1	3.96 ± 1.3*
IMA (AU)	87.1 ± 7.6	86.2 ± 7.4	108 ± 15*	87 ± 7.1	104 ± 10*	87.7 ± 6.3	80 ± 6.5*
cTnI (ng/L)	9 ± 7	9 ± 5	9 ± 4	8 ± 3	30 ± 9*	8 ± 10	8 ± 5
BNP (ng/L)	18 ± 11	24 ± 18	28 ± 9	24 ± 20	79 ± 14*	21 ± 12	24 ± 14
Hb (g/L)	15.3 ± 0.9	15 ± 0.3	14.8 ± 0.3	15.2 ± 0.2	16.4 ± 0.4*	15.2 ± 1.5	15.8 ± 0.9
Hematocrit (%)	45.5 ± 1.8	45 ± 0.9	45 ± 0.4	45 ± 0.7	46.3 ± 1.1*	44.9 ± 1.2	45.1 ± 1.4
Albumin (g/L)	38.5 ± 2	37 ± 2.4	37.6 ± 2.3	35 ± 1.9	39 ± 3*	38 ± 1.8	38 ± 1.8
Lactates (mmol/L)	1.1 ± 0.6	1.2 ± 0.3	1.4 ± 0.6	1.5 ± 0.6	1.9 ± 0.9*	1.1 ± 0.8	1 ± 0.7
Myoglobin (mg/L)	79 ± 6	80 ± 5	86 ± 6	70 ± 6	95 ± 12*	82 ± 3	79 ± 8
SpO_2_ (%)	98 ± 2	98 ± 0.7	82 ± 3.2*	99 ± 0.7	99 ± 0.9	98 ± 1.5	99 ± 1.9

The biomarker values are expressed as Mean ± SD in control subject (CNT), in apneic group before and after a single static apnea (APN‐S) and before and after a spearfishing competition (APN‐D) and SCUBA dive group before and after a single scuba dive (SCU‐D).

* Indicates significant difference at P < 0.05 between values before & after of one experimental condition (Wilcoxon test).

## Discussion

The main results show that the stress markers copeptin and IMA increased in apneic conditions but decreased in SCUBA conditions. Furthermore, markers of skeletal muscle injury and cardiac injury (cTnI) increased significantly in apneic dive conditions and they remained unchanged in SCUBA and static apneic conditions.

The SCU‐D and APN‐D situations combined elevated pressure and cold exposure. We hypothesized that these physiological stressors (cold exposure and elevated hydrostatic pressure) during SCUBA and apneic diving would induce elevated levels of copeptin and cortisol release. This was not seen with SCUBA diving and both markers increased during APN‐S testing at room temperature and normobaric pressure, suggesting that these factors alone did not induce the elevated levels of copeptin or IMA seen during apneic diving. These results are partly in agreement with a previous study showing that cold immersion had no significantly effect on ADH release (Jimenez et al. 2010). However, despite the long exposure, the combination of thermal insulation by the diving suit, summer water temperature (23°C), and activity‐related heat generation is likely to have resulted in a subthreshold level of thermal stress.

As previously described (Joulia et al. 2003), SpO_2_ decreased during the long‐duration static apnea (APN‐S: 140–450 sec) whose durations were sufficient to induce hypoxemia. In the dive situation (APN‐D), SpO_2_ measurement was delayed ~10 min by subject retrieval and sampling. As postapneic SaO_2_ reaches baseline in less than 2 min (Boussuges et al. 2011; Gargne et al. 2012), we have to infer marked hypoxemia in the APN‐D study from the apneic durations and levels of physical activity. However, the significant rise in IMA, a sensitive marker of hypoxia (Bar‐Or et al. 2001), strongly supports this inference.

Copeptin and IMA levels increased only during the two conditions associated with apnea (APN‐D and APN‐S). The fact that IMA is a sensitive marker of hypoxia (Bar‐Or et al. 2001), suggests that hypoxemia was the dominant physiological stressor. Competitive spearfishing (APN‐D) associates intense physical activity and breath‐holding, which together can induce hypoxemia even during short‐duration apnea (<1 min) (Joulia et al. 2002). Static apnea can also induce hypoxemia but it depends on the training level and only for longer duration (>2 min) apnea (Joulia et al. 2002). These results agree with studies showing that IMA (Nee et al. 2013; Joulia et al. 2015) and vasopressin (Baylis et al. 1977) are early and sensitive markers of hypoxemia. Since hypoxemia is the dominant physiological stressor, it is not surprising to observe an absence of increase (i.e., an absence of stress) and even a decrease in copeptin and IMA levels in SCU‐D that is associated with hyperoxia. Indeed, at depth, SCUBA divers breathe a gas mixture delivered at ambient hydrostatic pressure via a regulator. These hyperbaric conditions increase in oxygen partial pressure in proportion to inspired PO_2_ inducing hyperoxemia. However, the fact that a significant reduction in stress markers does occur with hyperoxemia suggests some level of residual “tone” in the stress marker response at rest and normoxic levels.

The lack of an increase in blood cortisol levels measured after the SCUBA dive is at odds with the increased saliva cortisol and decrease in cognitive performance observed by Pourhashemi et al. (2016). However, it agrees with the other studies showing no increase in stress hormone (Weist et al. 2012) or even a decrease (Lund et al. 1999) in blood cortisol level during SCUBA dives. This discrepancy between studies results could be partly explained by the level of expertise in SCUBA diving of the subjects. The SCUBA divers in our study were all well‐trained professional divers, whereas those of the study by Pourhashemi et al. (2016) were recreational divers much less experienced in diving. Furthermore, the hyperbaric O_2_ inspired during SCUBA diving might counterbalanced the effect of the cold immersion response by reducing the physiological stress level. Also contributing to this effect could be the raised hydrostatic pressure on the peripheral vasculature, which is known to lower adrenocortical hormone release (Weist et al. 2012). Regardless, cortisol appears to be a relatively insensitive marker in these situations as copeptin levels increased during APN‐S and decreased in SCU‐D without any changes in cortisol levels.

As acute hypoxia has been reported to increase the levels of stress hormone cortisol (Forslid and Augustinsson 1988) and growth hormone (Djarova et al. 1986), an unexpected result was the increase in cortisol levels with APN‐D but not APN‐S. Different factors might explain this lack of cortisol response with APN‐S. The duration of the apnea and hypoxemia was longer for the APN‐D as dives were repeated throughout the competition. Moreover, distance swimming in cold water (32 kms; 8.5 h and 18.5°C) induces cortisol release in experienced swimmers (Dulac et al. 1987). Finally, the APN‐D situation was a real championship competition and so the psychological stress and the intense physical activity could have contributed to cortisol release.

The increase in hematocrit and albumin levels indicates hemoconcentration, which has been reported previously for long‐lasting static immersion (Jimenez et al. 2010) and diving (Boussuges et al. 2011). In a spearfishing competition similar to that studied here, Boussuges et al. (2011) reported a mean weight loss of 1.5 kg mainly through dehydration and so the hemoconcentration measured in our study (APN‐D) could be an artifact of dehydration. Another explanation is splenic contraction in response to exercise (Laub et al. 1993) and the diving reflex, which is more pronounced in trained apnea divers (Richardson et al. 2009; Vigetun‐Haughey et al. 2015) can raise hematocrit several percent (Laub et al. 1993).

We observed an increase in cTnI and BNP levels in APN‐D that were not seen in SCU‐D or APN‐S, evidence of the more intense prolonged cardiac workload and hypoxemia in APN‐D. Indeed, electrocardiographic abnormalities (Marongiu et al. 2015; Zelenkova and Chomahidze 2016) or a decrease in stroke volume (Gargne et al. 2012) were previously described during apneic dive and attributed to the effect of pressure and/or hypoxemia. Hypoxemia without the increased workload in APN‐S was less stressful and not sufficient to raise cardiac markers. However, even if BNP is specific and sensitive for detecting early acute cardiac failure, despite the increase, the APN‐D levels remained within a normal rang and did not reach clinically significant levels (Feng et al. 2017), suggesting the presence of adaptation mechanisms in spearfishermen due to their high‐level of training.

Not surprisingly, we observed significant increases in lactate and myoglobin levels only in APN‐D conditions, the only condition of prolonged intense physical activity. However, considering the competitive situation, swimming distance, and repeated dynamic apneas, these increases seem mild, although in agreement with recent studies (Marongiu et al. 2015; Rodríguez‐Zamora et al. 2018).

In summary, we have identified increases in stress markers IMA and copeptin in hypoxic conditions, both hypoxia at rest and room temperature, and hypoxia during exercise and cold immersion associated with raised hydrostatic pressure. In hyperoxic conditions with cold and raised hydrostatic pressure conditions we identified a weaker decrease in these stress markers. Hypoxic stress, rather than cold, hydrostatic pressure or hyperbaric inspired gas is the physiological stressor that induces raised copeptin and IMA levels.

### Study limitations

We were limited by the real conditions in these aquatic environments and by the different populations that participate in these different activities. The exact matching of protocols and timing of sampling was not possible. Physiological parameters such as heart rate and temperature were not measured because the competition governing organization would not give permission. The levels of physical exercise of the apneic and SCUBA divers were not equivalent in duration or intensity. As the apneic divers participate at a higher level of competition they were significantly younger than the SCUBA divers. However, if exposed to hypoxia and the higher workload of the younger apneic group, the older SCUBA group could be expected to show greater stress responses. Thus, we have no reason to suspect that we underestimate the physiological stress of this level of SCUBA diving. As they stand, the results do reflect the real‐world physiology and hazards to which these groups are exposed.

## Conclusion and Perspectives

Despite differences in divers, depth, times, and protocols, this study compared different situations showing the reality of each diving activity. It showed an increase in stress markers (copeptin and IMA) in apneic diving condition but a decrease in the same markers in SCUBA diving condition. We conclude that hypoxia induced by apnea was the dominant trigger for the release of stress hormones and cardiac injury markers, whereas cold or/and hyperbaric exposures play a minor role.

Spearfishing is a very popular leisure activity around the world. It is regularly performed in winter or in cold country, sometimes associated with very strong conditions of currents or waves inducing a high level of physical activity. Despite their high‐level of training, our subjects demonstrated an increase in stress markers. Thus, one can expect an even larger increase in stress markers in less trained apnea divers. We thus suggest that all spearfishermen, and particularly noncompetitors, should be carefully screened for pre‐existing cardiac diseases before undertaking significant apneic maneuvers. Finally, since we only studied the effects of one single scuba dive, the effects of repetitive scuba dives remain unknown and should be investigate.

## Conflicts of Interest

None.
